# The cardiovascular response to passive movement is joint dependent

**DOI:** 10.14814/phy2.12721

**Published:** 2016-04-01

**Authors:** Keith J. Burns, Brandon S. Pollock, John McDaniel

**Affiliations:** ^1^ Department of Exercise PhysiologyKent State UniversityKentOhio; ^2^ Louis Stokes Cleveland Veterans Affairs Medical CenterClevelandOhio

**Keywords:** Blood flow, passive limb movement, transient hyperemic response, vascular health

## Abstract

The cardiovascular responses to passive limb movement (PLM) at the knee are well established, however, responses to PLM at other joints involving smaller muscle volume are unknown. To compare the cardiovascular responses to passive movement at other joints, 10 participants underwent a PLM protocol in which the wrist, elbow, ankle, and knee joints were passively extended and flexed at 1 Hz for 1 min. Heart rate (HR), mean arterial blood pressure (MAP), and arterial blood flow to that limb segment (BF) were measured and vascular conductance (VC) was calculated for a 30‐sec baseline period and for 3‐sec intervals throughout PLM protocols. PLM of the knee and elbow resulted in significant increases in BF and VC from baseline values with peak values 180% (*P* < 0.001) greater than baseline. PLM of the elbow resulted in significant increases in BF and VC from baseline values with peak values 109% and 115% (*P* < 0.001) greater than baseline, respectively. No changes in BF and VC were observed in the ankle and wrist. Furthermore, the greater increase in blood flow per limb segment volume in the thigh and upper arm (62.8 ± 36.5 and 55.5 ± 30.3 mL min^−1^ L^−1^, respectively) compared to the forearm and lower leg (23.6 ± 16.7 and 19.1 ± 10.3 mL min^−1^ L^−1^, respectively) indicates the limb volume is not solely responsible for the differences in the hyperemic responses. These data indicate that the use of PLM to assess vascular function or as a rehabilitation modality to maintain vascular health may be most appropriate for the muscles that span the elbow and knee.

## Introduction

In healthy persons, passive limb movement (PLM) induces a transient, yet substantial increase in limb blood flow and vascular conductance (Hayman et al. 2010; McDaniel et al. 2010a,b; Trinity et al. 2011). The hyperemic response to PLM is likely due to the activation of a series of peripheral mechanisms such as the skeletal muscle pump (Laughlin and Schrage 1999; Lutjemeier et al. 2005), mechanical distortion of arterioles (Clifford et al. 2006), release of vasodilators (Roseguini et al. 2010), and flow‐mediated dilation (Pyke et al. 2004). In addition, positive cardiovascular inotropic and chronotropic responses result from an increase in stroke volume, facilitated by increased venous return as well as increased neural feedback to the cardiovascular control center due to activation of mechanoreceptors (Craig 1995; Trinity et al. 2010). Taken together, these mechanisms ultimately result in a transient increase of cardiac output during PLM (McDaniel et al. 2010a).

Recently, investigators have sought to further partition the impact of central and peripheral regulators of blood flow in response to PLM to better understand whether these two mechanisms are obligatorily linked or work independently to create the hyperemic response. Trinity et al. (2010) and Venturelli et al. (2012, 2014) systematically investigated the influence of afferent feedback in healthy persons with the use of a fentanyl spinal block and the response to PLM in those spinal cord injuries. Additionally, Hayman et al. (2010) eliminated the effects of supraspinal cardiovascular control during PLM by employing individuals who had undergone a heart transplant. Together these studies have indicated that although a central hemodynamic response contributes to the hyperemic response during PLM, it is not obligatory and exemplified the importance of peripheral factors in inducing a hyperemic response. Previous investigators have also reported the importance of NO in mediating the hyperemic response during a bout of PLM (Trinity et al. 2015) and as such the magnitude of the transient hyperemic response induced by PLM has recently been suggested to serve as an indicator of vascular health and endothelial function (Mortensen et al. 2012; Trinity et al. 2012).

To the best of our knowledge, however, no previous investigations have examined the hyperemic and cardiovascular responses to PLM across any joint other than the knee. By altering the muscular structure and mass of the passively moved muscles there could be an attenuated hyperemic response due to reduced neural feedback to the cardiovascular control center and smaller skeletal muscle pump to increase venous return. However, mechanically induced vasodilation (Clifford et al. 2006) and release of vasodilators due to stretch/compression (Wozniak and Anderson 2009; Mortensen et al. 2012) should still result in a hyperemic response even in with reduced or absent central contributions. As such, the accurate evaluation of PLM applied to differing joints and limbs is of importance and could have a significant clinical impact. Therefore, the primary goal of this study was to compare the central and peripheral responses to PLM of the knee, ankle, elbow, and wrist joints. We hypothesized that PLM of the wrist, elbow, and ankle would result in a hyperemic response, however, this response would be reduced compared to that elicited by PLM of the knee.

## Methods

Healthy and recreationally active men (*n* = 5; age 26 ± 3 years; body mass 79.8 ± 11.3 kg; height 1.81 ± 0.08 m) and women (*n* = 5; age 24 ± 2 years; body mass 60.3 ± 6.7 kg; height 1.65 ± 0.08 m) volunteered to participate in this study. Participants were informed about the study and a written informed consent was obtained prior to their inclusion. The research protocol was reviewed and approved by the Kent State University Institutional Review Board.

### Experimental protocol

Participants refrained from eating and drinking caffeine for at least 3 h and refrained from exercise for 24 h prior to the experimental protocol. Initially, participants were required to lay supine for 20 min before the start of data acquisition and remained in this position for the duration of the protocol. The protocol consisted of one bout of passive movement across four different joints: wrist, elbow, ankle, and knee. For each bout of PLM a 30‐sec baseline measurement was followed directly by 1 min of PLM. The passive movement was conducted by a member of the research team moving the subject's joint through a physiological range of motion at a rate of 1 Hz. While we aimed to have a 90° range of motion for all four joints, the muscle and structural anatomy of the ankle and wrist slightly restricted movement and limited the range of motion. Specifically, the ankle joint was moved in a plantar‐flexion fashion from 90° to 135°. The wrist, elbow, and knee joints were moved from a joint angle of 0–90°. To limit any potential carryover between joints there was a 10‐min recovery period between bouts of PLM and heart rate was checked to ensure a return to baseline values. To limit the centrifugal and gravitational forces that act on the blood during movement of the elbow and knee, 1 min before the start of the passive movement a blood pressure cuff was placed distally to the joint and inflated to 250 mmHg, limiting blood flow to the tissue distal of the cuff placement (Hayman et al. 2010; McDaniel et al. 2010a,b; Ives et al. 2013). The cuff remained inflated for the entire duration of the elbow and knee protocols. For the wrist and ankle protocols no cuff was used because of the limited muscle mass distal to these joints. Cadence was kept consistent during all four protocols with the aid of a metronome. To avoid a startle reflex and active resistance or aiding to the passive movements, participants were given a light touch cue several seconds before the start of the passive movement. All movements were performed on the joints on the right side of the body.

### Measurements

#### Limb segment volumes

The volumes for each limb segment were calculated, as previously described (Lawrenson et al. 2003), based on limb segment circumference (three sites: distal, middle, and proximal), limb segment length, and limb segment skinfold measurements (Jones and Pearson 1969).

#### Arterial blood flow

Simultaneous measurements of arterial blood velocity and vessel diameter were performed during baseline and throughout the passive movement with a Logic 7 ultrasound system (General Electric Medical Systems, Milwaukee, WI) equipped with a linear array transducer (M12L) operating at a frequency of 14 MHz (ultrasound) and 5 MHz (Doppler). During the wrist, elbow, ankle, and knee protocols, arterial blood flow was measured in the brachial, axillary, popliteal, and common femoral artery, respectively. These locations provided the researchers with the most distal measurement of blood flow going to the muscles that were being lengthened and shortened. All blood velocity measurements were obtained with the probe appropriately positioned to maintain an insonation angle of less than or equal to 60°. Arterial diameter was determined at a perpendicular angle along the central axis of the scanned vessel. Mean blood velocity (*V*_mean_) values and arterial diameter were then automatically calculated using commercially available software installed on the GE logic 7 (General Electric Medical Systems, Milwaukee, WI) (Logic 7). Blood flow in the respective arteries were calculated as: blood flow = *V*_mean_*π(vessel diameter/2)^2^ × 60, where blood flow is calculated as milliliters per minute.

#### Central variables

Mean arterial pressure (MAP) was recorded with a Nexfin HD (BMIEYE B.V., Amsterdam, Netherlands) noninvasive beat‐by‐beat blood pressure monitor incorporated with an automated system for physiological calibration (Physiocal, BMIEYE). The finger cuff electrode was placed on the mid‐phalanx of the hand not involved with PLM. Heart rate (HR) was determined with the use of a 3‐lead ECG running through a BioAmp acquisition box (AD Instruments, Colorado Springs, CO). Vascular conductance (VC) was calculated as: VC = limb blood flow/MAP.

### Data acquisition and analysis

Throughout the protocols, MAP and HR signals underwent A/D conversion and were simultaneously acquired (200 MHz) using commercially available data acquisition software (Lab Chart 8 Pro; AD Instruments). Before the start of the passive protocols, values for each dependent variable were collected for 30 sec and averaged into one baseline value. During the passive protocols data were averaged into 3‐sec bins throughout each 60‐sec PLM protocol. To better explain the mechanisms for the hyperemic response, the values reported for each variable represent the value at the time in which peak blood flow occurred, not necessarily the peak value achieved during the PLM bouts as the peak values for all variables often do not temporally align. Relative change (% from baseline) was then calculated for all variables. In addition, the change in blood flow, from baseline to peak, was normalized for limb segment volume.

The dependent variables assessed were BF, HR, MAP, and VC across all conditions. Statistical analysis was performed using SPSS software (SPSS version 17; SPSS Inc, Chicago, IL). Initial three‐way repeated measures ANOVA (gender × limb × time) indicated no influence of gender on any of the variables, therefore data from males and females were grouped together for all subsequent analysis. Two‐way repeated measures ANOVA were used to determine if there was a significant main effect of time or limb or an interaction (limb × time) on blood flow and VC. If there was a significant main effect of time, simple contrasts (comparison of baseline to each 3‐sec average) were utilized to determine which of the 3‐sec bins were significantly different from baseline. Finally, if there was a significant interaction (limb × time), paired sample *t*‐tests were used to determine if the peak absolute and relative change (% change from baseline) were different between limbs. Paired samples *t* tests were utilized to determine if HR and MAP at peak blood flow was significantly different from baseline. Paired samples *t* test were also utilized to determine if the change in blood flow relative to limb segment volume was different between limbs. All values are presented as mean ± SD.

## Results

### Limb volume

Limb segment volumes for forearm, upper arm, lower leg, and thigh were 0.87 ± 0.19, 1.55 ± 0.52, 2.55 ± 0.58, and 6.93 ± 0.98 L, respectively. All limb segments volumes were statistically different from each other (*P* < 0.001).

Two‐way repeated measures ANOVA (limb × time) showed a significant interaction for BF (*P* < 0.001), HR (*P* = 0.003), and VC (*P* < 0.001), but not for MAP (*P* = 0.98).

### Heart rate

**Figure 1:**
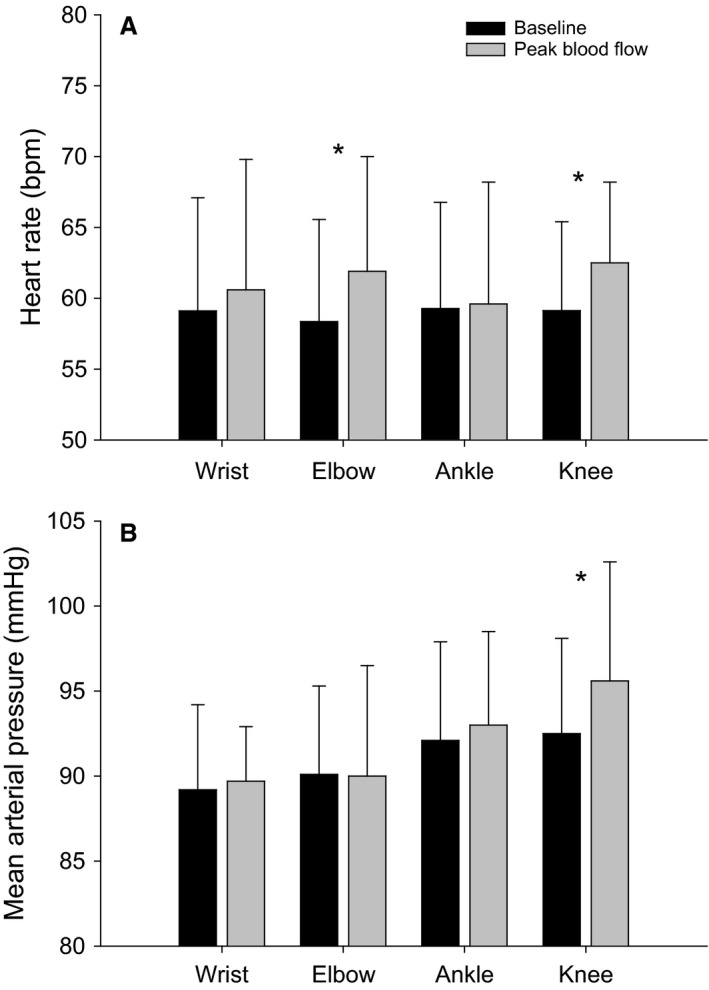
HR (A) and MAP (B) values during baseline and at peak blood flow. HR became significantly elevated during PLM of the elbow and knee while MAP became elevated during PLM of the knee only.

Significant increases in HR were observed at peak blood flow during passive movement of the elbow (58.4 ± 7.2 to 61.9 ± 8.1 bpm, *P* = 0.003) and knee (59.1 ± 6.2 to 62.5 ± 5.7 bpm, *P* = 0.007), both equaling a 6% change (Fig. 1A). There were no significant increases in HR during PLM of the wrist (*P* = 0.45) or ankle (*P* = 0.64).

### Mean arterial pressure

The PLM protocols of the wrist, elbow, ankle, and knee resulted in changes in MAP of 0.6%, −0.2%, 1.0%, and 3.3%, respectively, with the only significant difference in MAP occurring with passive movement of the knee (*P* = 0.039) (Fig. 1B).

### Blood flow

**Figure 2:**
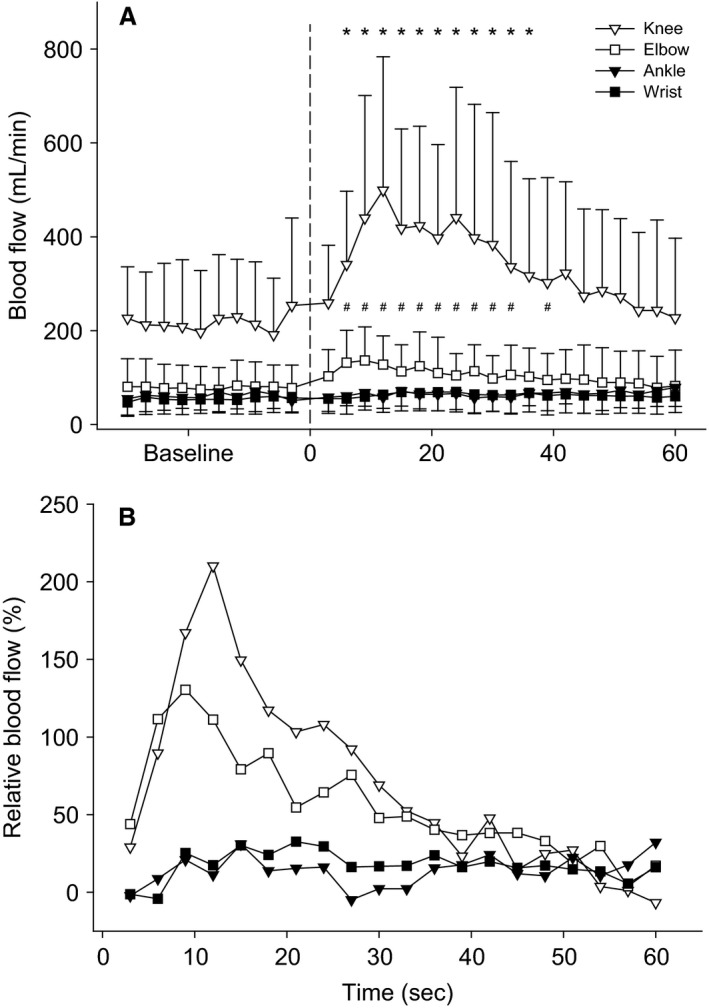
Blood flow (mL min^−1^) across 30 sec of baseline followed by 60 sec of passive limb movement. PLM of the knee resulted in significant increases in blood flow from baseline values for seconds 3–36 for the knee (*) and 3–39 for the elbow (#). (B) The relative changes in blood flow after normalizing to baseline values.

**Figure 3:**
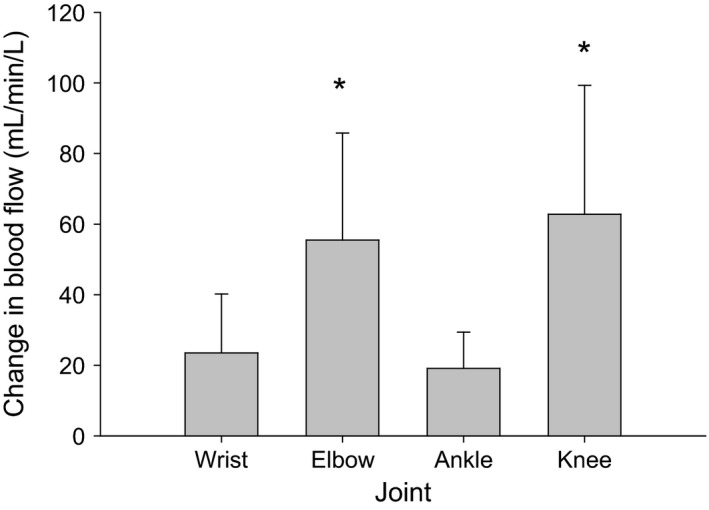
Change in blood flow relative to limb volume. *Indicates a significant difference from wrist and ankle. No other comparisons between joints were significant (*N* = 9).

Femoral artery blood flow increased from 225 ± 126 mL min^−1^ during baseline to an average peak 627 ± 273 mL min^−1^ (Δ180%) during PLM of the knee. A significant hyperemic response from baseline values was observed from seconds 3 to 36 of PLM before blood flow returned to baseline levels (Fig. 2A). PLM protocol of the elbow elicited an increase in axillary blood flow from 79 ± 47 mL min^−1^ during baseline to a peak of 167 ± 84 mL min^−1^ (Δ109%). A significant hyperemic response was observed from seconds 3 to 39, sans seconds 33 to 36, before returning to baseline (Fig. 2A). The average peak increase in femoral blood flow of 403 ± 244 mL min^−1^, which occurred during seconds 9–12 of the PLM about the knee, was significantly greater than the 88 ± 45 mL min^−1^ average peak increase in axillary blood flow, which occurred during seconds 6–9 of PLM about the elbow. Paired sample *t* tests indicated that the peak relative change in BF between the knee and elbow were not statistically different (Fig. 2A). Baseline blood flow values for the brachial and popliteal arteries were 52 ± 31 and 61 ± 27 mL min^−1^ and in contrast to PLM of the knee and elbow did not significantly increase during PLM of the wrist and ankle joints, respectively (Fig. 2A and B). Paired *t* tests indicate that the blood flow relative to the limb segment volume in the upper arm and thigh (55.5 ± 30.3 and 62.8 ± 36.5 mL L^−1^ min^−1^, respectively) were not different from each other (*P* = 0.533), but both were greater than blood flow relative to limb segment volume in the forearm and lower leg (23.6 ± 16.7 and 19.1 ± 10.3 mL L^−1^ min^−1^, respectively) (*P* ≤ 0.017). Blood flow relative to limb segment volume was not different between the lower leg and forearm (*P* = 0.494) (Fig. 3).

### Conductance

**Figure 4:**
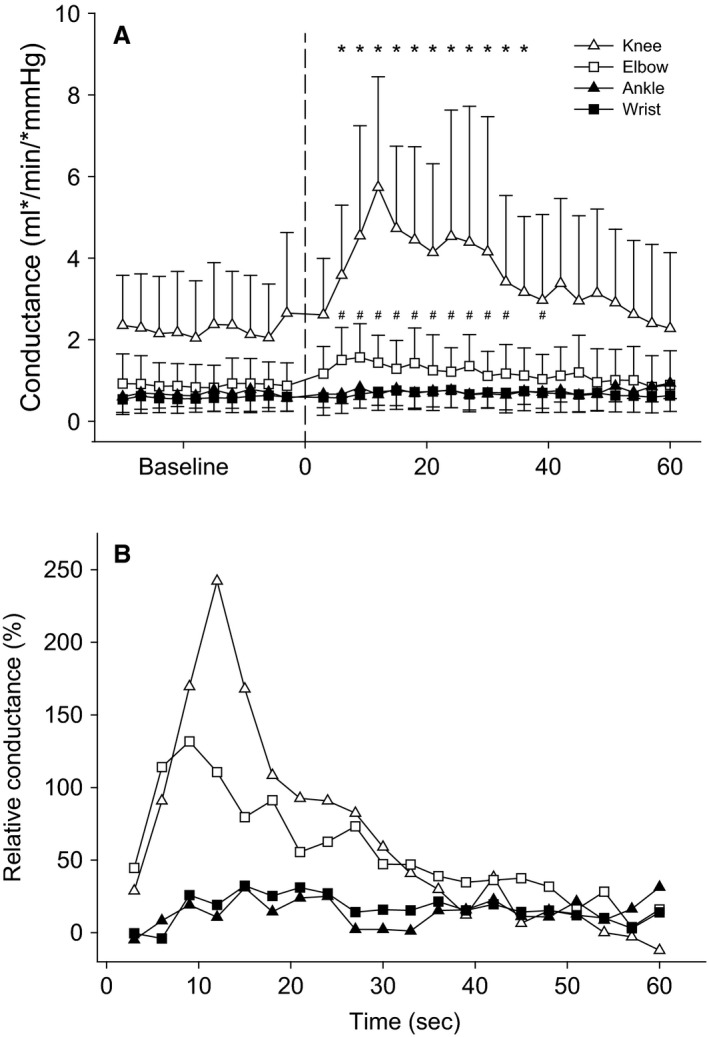
Conductance (mL min^−1^ mmHg^−1^) across 30 sec of baseline followed by 60 sec of passive limb movement (PLM). (A) PLM of the knee resulted in significant increases in conductance from baseline values for seconds 3–36 for the knee (*) and 3–39 for the elbow (#). (B) The relative change in conductance during the 60 sec of PLM after normalizing to baseline values.

At baseline, the conductance in the femoral artery was 2.3 ± 1.3 mL min^−1^ mmHg^−1^. During PLM of the knee a significant increase in conductance was observed for seconds 3–36 and peaked at 6.4 ± 2.8 mL min^−1^ mmHg^−1^ (Δ180%) before returning to near baseline values. Baseline conductance in the axillary was 0.88 ± 0.54 mL min^−1^ mmHg^−1^, was significantly elevated from 3 to 39 seconds of PLM, and peaked at 1.9 ± 0.9 mL min^−1^ mmHg^−1^ (Δ115%). The average peak increase in VC 4.1 ± 1.0 mL min^−1^ mmHg^−1^ for the knee was significantly greater than the average peak increase in 1.0 ± 0.5 mL min^−1^ mmHg^−1^ observed for the elbow. Paired samples *t* tests revealed no statistical difference between peak relative changes in VC during PLM of the knee and elbow. Similar to blood flow, no significant increase in VC was observed during PLM of the wrist and ankle joints (Fig. 4A and B).

## Discussion

The present study is novel in that it is the first to examine the differences in the central and peripheral hemodynamic responses associated with PLM across joints other than the knee. Specifically, we compared the absolute and relative changes in blood flow, conductance, heart rate, and mean arterial pressure during a 1‐min bout of 1 Hz PLM of the knee, ankle, elbow, and wrist. These data indicate that PLM of the knee and elbow, but not wrist or ankle, resulted in significant increases in blood flow, conductance, and heart rate. Furthermore, the changes in blood flow relative to limb segment volume indicate that the magnitudes of the hyperemic responses were not entirely based on muscle volume. These data partially agree with our hypothesis in that we expected a reduced, but not absent, hyperemic response with PLM applied to the wrist and ankle. The hyperemic responses observed with passive movement of the elbow, but not ankle and wrist, indicate that the previously proposed use of PLM at the knee to (1) assess vascular function or (2) use as a rehabilitation tool to illicit hyperemic responses to maintain vascular function and tissue perfusion in nonmobile populations, may extend to movement at the elbow but not necessarily wrist and ankle.

### Central regulators of blood flow

Previous literature has indicated the transient hyperemic response during PLM results from several mechanisms. Activation of type III muscle afferents (Barron and Coote 1973; Trinity et al. 2010; Venturelli et al. 2012) results in neural feedback directed to the cardiovascular control center located in the medulla oblongata resulting in a positive chronotropic response (Amann et al. 2010). During dynamic exercise, the exercise pressor reflex (EPR) is mediated by exercise intensity and muscle mass, with a larger EPR activation observed at greater exercise intensities and with the involvement of greater muscle mass within the lower body (Freund et al. 1978; Iwamoto and Botterman 1985; Iellamo et al. 1999). Although the PLM model as a whole is void of the type IV afferent signals, the marked reduction in the hyperemic response in the wrist and ankle, compared to the knee and elbow, is likely partially due to reduced muscle mass about those joints, reduced total afferent type III feedback to the cardiovascular control center and subsequently reduced or no increase in HR. However, this is not the entire explanation as the lower leg had a greater volume than the upper arm, but a reduced HR response. The differences in sensitivity of the proprioceptive systems across joints (Hall and McCloskey 1983) may explain this discrepancies.

Although stroke volume was not recorded during the present investigation, the influence of the skeletal muscle pump on stroke volumes has been extensively studied and reviewed (Clifford and Hellsten 2004; Tschakovsky and Sheriff 2004) and has been shown to play a role in the cardiovascular responses to PLM. Specifically, McDaniel et al. (2010a) reported that inflating a cuff placed around the inguinal region of the passively moved limb, thereby occluding venous return, resulted in a significant decrease in stroke volume and subsequently cardiac output when compared to the noncuffed condition. In the present study, it is likely that the smaller muscles volume about the ankle, wrist, and even the elbow represented a smaller skeletal muscle pump thereby reducing venous return and subsequent stroke volume compared to the muscle mass of the thigh.

### Peripheral

In addition to the changes in SV and CO, previous investigators have reported the hyperemic responses during PLM of the knee can be attributed to peripheral factors. PLM in subjects with neural blockade (Trinity et al. 2010), heart transplants (Hayman et al. 2010), and spinal cord injuries (Venturelli et al. 2012, 2014) all indicate that the hyperemic response can be completely or partially preserved even with no increase in cardiac output. Furthermore, investigators have reported the importance of peripheral local factors in modulating the hyperemic response via intra‐arterial infusion of NG‐monomethyl‐l‐arginine (l‐NMMA) which inhibits nitric oxide synthase (Mortensen et al. 2012; Trinity et al. 2012, 2015), and results in decreases in the bioavailability of the potent vasodilator nitric oxide (NO). Specifically, the infusion of l‐NMMA reduced the overall hyperemic response to PLM in the femoral artery by 80% compared to control conditions, despite the fact HR, SV, CO, and MAP had similar changes. Accordingly, despite not observing an increase in HR, we were surprised to observe no hyperemic response in the popliteal or brachial artery during PLM of the ankle and wrist, respectively. We expected peripheral factors such as mechanically induced vasodilation (Clifford et al. 2006) and release of vasodilators following stretch/compression (Wozniak and Anderson 2009; Mortensen et al. 2012) would increase conductance and ultimately contribute to some increase in blood flow. Although the specific mechanisms for this compression induced vasodilation are not clearly established, they appear to be both endothelial and myogenic in nature (Clifford et al. 2006) with nitric oxide, prostaglandin, and/or endothelium‐derived hyperpolarizing factors proposed as the endothelial mechanisms and mechanosensitive ion channels or integrins proposed as the myogenic mechanisms (Clifford and Tschakovsky 2008). Further research is needed to determine which of these potential mechanisms may be absent in the more distal muscles, possibly preventing the hyperemic response.

### Vascular function

Recently it has been proposed that the magnitude of hyperemic response elicited by PLM of the knee could be used as a means to assess vascular health as large portion of the hyperemic response appears to be highly dependent on NO release (Trinity et al. 2012). Additional studies have also reported the ability of PLM to upregulate numerous angiogenic and vasodilatory factors resulting in endothelial cell proliferation in skeletal muscle (Hellsten et al. 2008; Høier et al. 2010; Hoier et al. 2013). Although these data indicate a hyperemic response is produced in the axillary artery during PLM of the elbow, more research is required to determine the role of NO and the potential for using PLM of the elbow to assess vascular function in the upper arm, potentially as a replacement for the more traditionally used flow‐mediated dilation protocols. Thus, the present study does not dispute these previous findings but it does imply an impetus that discretion may need to be taken if PLM is used as a tool to assess whole body vascular health or prompt angiogenic processes because the magnitude of a local hyperemic responses vary between limbs and may in fact be absent altogether.

While there was no significant increase in net blood flow (i.e., antegrade − retrograde) during ankle and wrist PLM, there was noticeable increase in both the retrograde and antegrade blood flow of the popliteal and brachial arteries (not reported). This agrees with previous reports that indicate following the return of net blood flow to baseline during PLM, antegrade and retrograde blood flow remain elevated (McDaniel et al. 2010a) While the influence of retrograde blood flow on vascular function is currently debated (Green et al. 2002, 2005; Thijssen et al. 2009), the increased total shear stress (antegrade + retrograde) observed during these PLM protocols may represent adequate potency need to illicit vascular adaptations.

### Limitations

While a range of motion of 90° was desired for all joints, the wrist and specifically the ankle joint exhibited a marked reduction in flexibility compared to the elbow and knee. One could contend this smaller range of motion, despite the fact that it was near maximal physiological range of motion for those two joints, is responsible for the near absent responses observed in the ankle and wrist to PLM and therefore similar joint angle excursion should have been utilized. However, having similar joint angle excursions does not mean the range in muscle fiber shortening and lengthening is similar. Specifically, the architecture of the muscles that span those joints including the pennation angle of the fibers and moment arm (distance between muscle insertion and joint center) mediates the relationship between joint angle excursion and changes in muscle fiber length. In addition, fascicle lengths are very different for muscles at different joints (Paul 2001) and detection thresholds of muscle spindles are not dependent on the joint angular range of motion, rather the proportional length change of the muscle fascicles that operate about that joint (Refshauge et al. 1995, 1998). Furthermore, it is likely that the magnitude of the mechanical compression on the vessels is not dependent on absolute changes in muscle length, rather changes in muscle length relative to physiological ROM. For example, imagine the ROM about a joint is 90° and limited by the flexibility of the muscle‐tendon units that span that joint. After 1 month of flexibility training, the ROM about that joint has increased to 130°. The stress and therefore compression of the vessels across the original 90° ROM will likely be much less following the month of flexibility training. Thus, utilization of similar joint angles also has its own set of limitations. Considering the use of PLM for rehabilitation, we decided to simply use joint ROM similar to the physiological ROM rather than an absolute ROM across all joints.

## Conclusions

Passive movement of the elbow and knee resulted in similar magnitude and time course changes in blood flow, conductance, and heart rate. Contrarily, passive movement of the ankle and wrist did not result in any changes in blood flow, conductance, and heart rate. These differences are not explained by limb volume as the upper arm had a smaller limb volume but a greater hyperemic response compared to the lower leg. Furthermore, the equal hyperemic responses between the thigh and upper arm, when expressed relative to limb segment volume, indicate that PLM may be an alternative method to assess vascular endothelial function in the upper arm similar to its recent proposed use to assess endothelial function in the thigh. Future research is required to determine the role of NO in passive movement of the elbow and determine the mechanisms responsible for lack of hyperemic response in the forearm and ankle.

## Conflict of interest

None declared.
